# Draft Genome Sequence of Marine Flavobacterium *Jejuia pallidilutea* Strain 11shimoA1 and Pigmentation Mutants

**DOI:** 10.1128/genomeA.01236-14

**Published:** 2014-12-04

**Authors:** Naoki Takatani, Masato Nakanishi, Pedro Meirelles, Sayaka Mino, Wataru Suda, Kenshiro Oshima, Masahira Hattori, Moriya Ohkuma, Masashi Hosokawa, Kazuo Miyashita, Fabiano L. Thompson, Ako Niwa, Toko Sawabe, Tomoo Sawabe

**Affiliations:** a Faculty of Fisheries Sciences, Hokkaido University, Hakodate, Japan; b Laboratory of Microbiology, Institute of Biology and Solid Action on Globalization and Environment, COPPE, Federal University of Rio de Janeiro, Rio de Janeiro, Brazil; c Center for Omics and Bioinformatics, Graduate School of Frontier Sciences, The University of Tokyo, Kashiwa, Japan; d Microbe Division/Japan Collection of Microorganisms, RIKEN BioResource Center, Ibaraki, Japan; e Department of Food and Nutrition, Hakodate Junior College, Hakodate, Japan

## Abstract

Here, we present the draft genome sequence of a novel carotenoid 2′-isopentenylsaproxanthin producer, *Jejuia pallidilutea* strain 11shimoA1, isolated from the surface of seaweed in Japan, and the ethyl methanesulfonate-induced pigmentation mutants. This genomic information will help to not only elucidate the 2′-isopentenylsaproxanthin biosynthetic pathway but also understand the evolution of flavobacteria.

## GENOME ANNOUNCEMENT

The family *Flavobacteriaceae* was established for grouping a diverse array of bacteria showing Gram-negative, aerobic, nonmotile pigmented bacteria that show yellow to orange colored colonies (1) and includes more than 114 genera (http://www.bacterio.net). One of the genera, *Jejuia*, was proposed in 2009 for nonmotile, yellow, nondiffusible pigment producers in seawater and consists of a single species *J. pallidilutea* (2, 3). More recently, Takatani et al. (4) have identified a strain 11shimoA1 isolated from the surface of seaweed that produces not only zeaxanthin but also a new monocyclic carotenoid, 2′-isopentenylsaproxanthin. The amount of 2′-isopentenylsaproxanthin production increased under alkaline conditions (4). However, carotenoid synthetic pathways of the 2′-isopentenylsaproxanthin have not been resolved yet.

The genome sequences of *J. pallidilutea* 11shimoA1 (JCM 19538) and the pigmentation mutants induced by ethyl methanesulfonate were sequenced with the Illumina HiSeq (Illumina) and Ion PGM systems (Life Technologies, Carlsbad, CA), respectively. The genome sequence of 11shimoA1 was *de novo* assembled using Velvet version 1.2.08. Those mutants were *de novo* assembled using Newbler version 2.8. The annotation and genome analysis were performed by RAST (Rapid Annotation Subsystem Technology) (5). The sizes of the draft genome of *J. pallidilutea* 11shimoA1, the pigment-deficient mutant A1W, and the red-pigmented mutant A1R were 3,805,351 bp, 3,773,436 bp, and 3,770,805 bp, comprising 98, 75, and 87 contigs with G+C contents of 33.9%, 33.9%, and 33.8%, respectively. These redundancies were 779, 20, and 17, and *N*_50_ contig lengths were 244,792 bp, 135,822 bp, and 112,803 bp, respectively. Putative coding sequences (CDS) were 3,328, 4,046, and 4,232; rRNA sequences were 3, 3, and 3; tRNA sequences were 36, 34, and 35 for strains 11shimoA1, A1W, and A1R, respectively. The strain 11shimoA1 possessed a set of carotenoid biosynthesis gene clusters (phytoene dehydrogenase, phytoene synthase, β-carotene hydroxylase and lycopene β cyclase), but the other set of phytoene dehydrogenase and lycopene elongase was located on a different locus of the genome. It is suggested that these genes may be responsible for producing zeaxanthin and 2′-isopentenylsaproxanthin. In addition, genome comparisons between the genomes of 11shimoA1 and A1W revealed a frameshift in the phytoene dehydrogenase gene of the A1W genome. These strains have been deposited in the Japan Collection of Microorganisms as JCM 19301 (A1W) and JCM 19302 (A1R), respectively.

### Nucleotide sequence accession numbers.

The genome data have been deposited in DDBJ/EMBL/GenBank under the accession numbers BBNY01000001 to BBNY01000098, BBNR01000001 to BBNR01000075, and BBNS01000001 to BBNS01000087 for *Jejuia pallidilutea* strains 11shimoA1, A1W, and A1R, respectively.
